# Effects of simvastatin, rosuvastatin and pravastatin on soluble fms-like tyrosine kinase 1 (sFlt-1) and soluble endoglin (sENG) secretion from human umbilical vein endothelial cells, primary trophoblast cells and placenta

**DOI:** 10.1186/s12884-016-0902-3

**Published:** 2016-05-20

**Authors:** Fiona C. Brownfoot, Stephen Tong, Natalie J. Hannan, Roxanne Hastie, Ping Cannon, Tu’uhevaha J. Kaitu’u-Lino

**Affiliations:** Translational Obstetrics Group, Department of Obstetrics and Gynaecology, University of Melbourne, Mercy Hospital for Women, 163 Studley Road, Heidelberg, 3084 Victoria Australia

**Keywords:** Preeclampsia, Pregnancy, Statins, sFlt-1, Soluble endoglin

## Abstract

**Background:**

Preeclampsia is associated with the placental release of soluble fms-like tyrosine kinase 1 (sFlt-1) and soluble endoglin (sENG). These anti-angiogenic factors cause hypertension and multi-organ injury. Pravastatin decreases placental secretion of sFlt-1 in vitro and is currently being examined in clinical trials as a potential treatment for preeclampsia. However, it is possible that different classes of statins may be more potent at decreasing sFlt-1 secretion. We compared the relative potency of three different generations of statins on sFlt-1 and sENG secretion from human endothelial cells, trophoblast cells, and placenta explants.

**Methods:**

We performed functional experiments using primary human umbilical vein endothelial cells, trophoblast cells and preterm preeclamptic placental explants to assess the affect of simvastatin, rosuvastatin and pravastatin on sFlt-1 and sENG secretion and compared the relative potency of each statin at reducing these factors (Inhibitory Concentration 50). Furthermore we assessed the effect of each statin on the antioxidant and cytoprotective enzyme, heme-oxygenase 1.

**Results:**

All statins reduced sFlt-1 secretion from endothelial cells, trophoblasts and preterm preeclamptic placental explants. Simvastatin was the most potent inhibitor of sFlt-1 secretion from endothelial cells (IC 50 3.2 μM), trophoblast cells (IC 50 61.4 μM) and placental explants. Simvastatin was 28 times and 3 times more potent at reducing sFlt-1 secretion from endothelial cells and 85 times and 33 times more potent at reducing sFlt-1 secretion from trophoblast cells than pravastatin or rosuvastatin respectively.

All statins increased sENG secretion from endothelial cells however did not change secretion from placental explants.

While all statins up-regulated heme-oxygenase 1 in endothelial cells, only simvastatin up-regulated its expression in placenta from patients with preterm preeclampsia.

**Conclusion:**

Simvastatin may be a more potent inhibitor of sFlt-1 secretion from endothelial cells, trophoblast cells and placenta from women with preterm preeclampsia than either pravastatin or rosuvastatin.

## Background

Preeclampsia is associated with the placental release of soluble fms-like tyrosine kinase 1 (sFlt-1) [1] and soluble endoglin (sENG) [2] into the maternal circulation leading to hypertension, proteinuria and multi-system organ injury [3–7]. Currently there is no medical treatment that stabilises the disease pathophysiology and delivery, often at preterm gestations, is required to stop maternal disease progression [3, 4]. A therapeutic that stabilises the maternal disease process allowing preterm pregnancy continuation, or reducing progression to severe disease at term, would be beneficial [8].

Pravastatin has been proposed as a therapeutic candidate for preeclampsia [9–14]. It has been shown to reduce the preeclamptic phenotype in 4 different animal models of the disease [11–14]. We recently published a case series showing administration of pravastatin may stabilise biochemical features of the disease in 3 out of 4 cases of preterm preeclampsia [10]. We, and others have also shown pravastatin [10] and simvastatin [9] reduce sFlt-1 secretion from human placental tissue. This is a desirable feature of a preeclampsia therapeutic as sFlt-1 is a likely pathogenic protein of the disease [1]. Firstly, sFlt-1 is elevated in patients presenting with preeclampsia [15] compared to levels in normal pregnancy and secondly, when injected or overexpressed in mice and rats, hypertension and proteinuria occur [1, 2]. Importantly, when sFlt-1 levels were reduced in preeclamptic patients by plasma apheresis, there was a possible stabilisation of disease [16].

Pravastatin [10] and simvastatin [9] have both been shown to exert an effect on sFlt-1 secretion by inhibiting HMG CoA reductase, a key enzyme in the cholesterol synthesis pathway. There are many statins and they are divided into three different generations based on their relatively potencies in reducing low density lipoproteins, a surrogate marker for their inhibition of HMG CoA reductase [17]. It is therefore conceivable that more potent inhibitors of HMG CoA reductase, might produce greater reductions in sFlt-1 secretion.

Pravastatin is a first generation statin and in fact one of the least potent inhibitors of HMG CoA reductase [17]. Simvastatin is a second generation statin and a more potent inhibitor, whilst rosuvastatin is the most potent inhibitor of HMG CoA reductase and sole third generation statin [17]. Whilst in vivo studies [11–14] and clinical trials [18, 19] have focused on pravastatin as a preeclampsia therapeutic, it is conceivable that simvastatin or rosuvastatin might have a more pronounced effect on decreasing sFlt-1 secretion.

We compared the potency of simvastatin, rosuvastatin and pravastatin on sFlt-1 and sENG secretion in vitro using human endothelial cells, trophoblast cells and placental explants obtained from women with preterm preeclampsia. We assessed whether statins induced the cytoprotective enzyme, heme-oxygenase 1.

## Methods

Ethics approval was obtained from the Mercy Health Human Research Ethics Committee and all women gave written informed consent prior to the collection and use of placenta and umbilical cord.

### Isolation and treatment of primary human umbilical vein endothelial cells (HUVECs)

Umbilical cords were collected from normal term placentas. The cord vein was infused with 10 ml (1 mg/ml) of collagenase (Worthington, Lakewood, New Jersey) and cells isolated as previously described [20]. Cells were cultured in M199 media (Life Technologies, Victoria, Australia) containing 10 % FCS (Sigma, St Louis, United States), 1 % antibiotic-antimycotic (Life Technologies) and 20 μg/ml endothelial cell growth factor (ECGS) (Sigma) and 100 μg/ml heparin (Sigma) and used between passages 2 to 4. Cells were plated at 24,000 /cm^2^ and treated at 80 % confluency with 0, 1, 2 or 5 μM simvastatin or rosuvastatin and 0, 2, 5 or 200, μM pravastatin for 24 h. Conditioned media was collected for assessment of sFlt-1 and sENG secretion and cell lysates collected for RNA extraction.

### Isolation and treatment of primary human trophoblast cells

Term placentas were collected from women having elective caesarean sections. Human trophoblasts were isolated as previously described [20, 21]. Primary trophoblasts were cultured in DMEM high Glutamax (Life Technologies) containing 10 % FCS and 1 % antibiotic-antimycotic (Life Technologies) on fibronectin (10 μg/mL; BD Biosciences, New South Wales, Victoria) coated plates. Cells attached over 24 h before being washed in PBS [20]. Cells were treated with 0, 5, 50, 100 μM simvastatin, 0, 100, 200, 300 μM rosuvastatin or 0, 20, 200, 2000 μM pravastatin for 24 h under 8 % O_2_ and 5 % CO_2_ at 37 °C. Conditioned media was collected to assess sFlt-1 secretion.

### Cell viability assay (MTS assay and calcein stain)

Cell viability assay were performed using CellTiter 96-Aquesous One solution (Promega, Madison WI) or calcein stain (Merk Millipore, Darmstadt, Germany) according to the manufacturer’s instructions. Fluostar omega fluorescent plate reader (BMG labtech, Victoria, Australia) was used to detect fluorescence (quantify adhesion).

### Placental explant culture

Human placental tissue was collected from three women with severe early onset preeclampsia (delivered at ≤34 weeks gestation). Preeclampsia was defined using the 2013 American College of Obstetricians and Gynecologists (ACOG) guidelines: the presence of hypertension >140/90 on two occasions 4 h apart and any of the following: proteinuria >300 mg/day, renal insufficiency, impaired liver function, thrombocytopenia or visual disturbance [22].

Villous explants were prepared as previously described [10, 20] and cultured in DMEM high glutamax (Life Technologies) containing 1 % antibiotic-antimycotic (Life Technologies) and 10 % fetal calf serum (FCS) (Sigma). After 24 h placental explants were treated with 0, 100 μM simvastatin, 0, 100 μM rosuvastatin or 0, 2000 μM pravastatin (Sigma) for 72 h under 8 % O_2_ and 5 % CO_2_ at 37 °C. To assess sFlt-1 and sENG secretion, protein levels were normalized against wet placental explant weights. Tissue was collected for RNA extraction.

### ELISA analysis

Concentrations of sFlt-1 and sENG were measured in conditioned cell/tissue culture media using the DuoSet VEGF R1/Flt-1 kit (R&D systems by Bioscience, Waterloo, Australia) and a DuoSet Human Endoglin CD/105 ELISA kit (R&D systems) according to manufacturer’s instructions.

### RT-PCR

RNA was extracted from placental explants and HUVECs using an RNeasy mini kit (Qiagen, Valencia, CA) and quantified using the Nanodrop ND 1000 spectrophotometer (NanoDrop technologies Inc, Wilmington, DE). 0.2 μg of RNA was converted to cDNA using Superscript VILO cDNA synthesis kit (Life Technologies) as per manufacturer guidelines.

A taqman gene expression assay was performed for *heme-oxygenase 1* (Life Technologies). Primer sequence for *heme-oxygenase 1* was forward primer 5′-GGAGGAGGAGATTGAGCACAACA-3′ and reverse primer 5′-AGCGGTACAGCTGCTTGAAC-3′. RT-PCR was performed on the CFX 384 (Bio-Rad, Hercules, CA) using FAM-labeled Taqman universal PCR mastermix (Life Technologies) with the following run conditions: 50 °C for 2 min; 95 °C for 10 min, 95 °C for 15 s, 60 °C for 1 min (40 cycles). Sybr gene expression assay for *sFlt-1 e15a* and *sFlt-1 i13* was used. Primers for *sFlt-1 e15a* (forward 5′-CTCCTGCGAAACCTCAGTG-3′ and reverse 5′-GACGATGGTGACGTTGATGT-3′) and sFlt-1 i13 (forward 5′-ACAATCAGAGGTGAGCACTGCAA-3′ and reverse 5′TCCGAGCCTGAAAGTTAGCAA-3′) were designed as previously described (Geneworks, South Australia, Australia) [23]. RT-PCR was performed using the following run conditions: 95 °C for 20 min; 95 °C for 0.01 min, 60 °C for 20 min, 95 °C for 1 min (39 cycles), melt curve 65 °C to 95 °C at 0.05 °C increments at 0.05 s.

All data were normalized to GAPDH as an internal control and calibrated against the average C_t_ of the control samples. GAPDH mRNA expression was stable in control compared to statin treatment groups. Results are expressed as fold change from control.

### Statistical analysis

Technical replicates were performed in triplicate for each experiment, with a minimum of three separate experiments repeated for each in vitro study. Data was tested for normal distribution and statistically analysed as appropriate. When three or more groups were compared a 1-way ANOVA (for parametric data) or Kruskal-Wallis test (for non-parametric data) was used. Post-hoc analysis was carried out using either the Tukey (parametric) or Dunn’s test (non-parametric). When two groups were analysed, either an unpaired *t*-test (parametric) or a Mann-Whitney test (non-parametric) was used. All data is expressed as mean ± SEM.

The IC50 values were determined by plotting the effect of the three statins on sFlt-1 secretion on the same graph. Doses of the statins were transformed and the log of the value recorded. The maximum, minimum and slope of the curve were shared between data sets and non-linear regression was performed. *P*-values <0.05 were considered significant. Statistical analysis was performed using GraphPad Prism 6 software (GraphPad Software, La Jolla, CA).

## Results

### Statin treatment of endothelial cells, primary trophoblasts and placental explants obtained from patients with preterm preeclampsia

We have previously reported the effect of pravastatin on endothelial cells, primary trophoblasts and preeclamptic placental explants [10]. Here we dose matched pravastatin to simvastatin and rosuvastatin and also included a dose that has an effect on sFlt-1, in order to perform a comparison of potency. The doses were chosen based on an ability to exert an effect on sFlt-1 whilst maintaining cell viability. Cell viability was maintained for all statins at the doses used in both placental and endothelial cells, except for 5 μM simvastatin treatment of endothelial cells, which reduced metabolic activity (a measure of cell viability) by 17 % (data not shown). Experiments using the three different statins were performed on the same tissues at the same time so comparisons could be made.

### Simvastatin is the most potent inhibitor of sFlt-1 secretion from primary endothelial cells

**Fig. 1:**
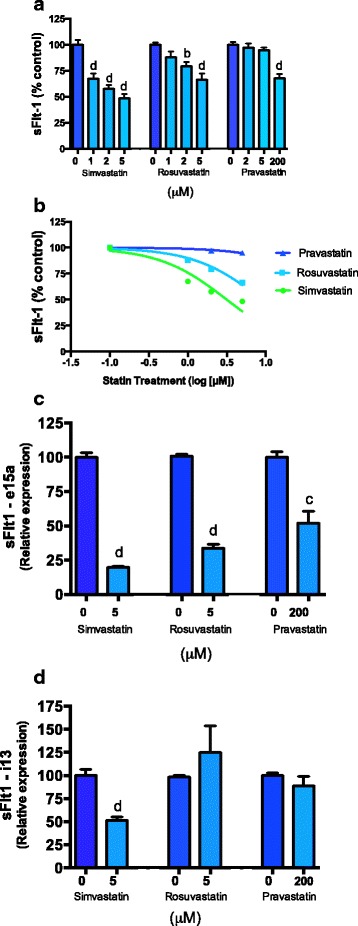
Effects of statins on sFlt-1 secretion and mRNA expression of isoforms *e15a* and *i13* expression in primary HUVECs. **a** Simvastatin (0, 1, 2, 5 μM) rosuvastatin (0, 1, 2, 5 μM) and pravastatin (0, 2, 5, 200 μM) reduce sFlt-1 secretion from primary HUVECs. **b** Inhibitory concentration of sFlt-1 at 50 % (IC50) for simvastatin, rosuvastatin and pravastatin was determined. **c** Pravastatin, simvastatin and rosuvastatin reduce *sFlt-1 e15a mRNA* expression from primary HUVECs. **d** Simvastatin reduces *sFlt-1 i13 mRNA* expression from HUVECs whilst pravastatin and rosuvastatin have no effect. Data represents *n* = 3 separate experiments and is expressed as mean ± SEM. *Dark blue bars* = control, *light blue bars* = statin treatments. b = *p* < 0.01, d = *p* < 0.0001

We first examined the effect of simvastatin, rosuvastatin and pravastatin on sFlt-1 secretion from primary human umbilical vein endothelial cells (HUVECs). All statins induced a significant dose dependent reduction in sFlt-1 secretion from primary HUVECs (Fig. 1A). Simvastatin was the most potent inhibitor of sFlt-1 secretion with an IC50 (concentration of the drug required to reduce secretion by 50 %) of 3.1 μM. This was 28 fold more potent than pravastatin (IC50 88.5 μM) and 3 fold more potent than rosuvastatin (IC50 8.9 μM) (Fig. 1B).

Next we explored the effect of the different statins on the expression of two isoforms of *sFlt-1*. sFlt-1 is comprised of a number of different splice variants that differ in sequence at the c-terminal region. The most abundant variant in preeclampsia is *sFlt-1 e15a* which is primate specific and accounts for >80 % of sFlt-1 secreted from the placenta [24]. *sFlt-1 i13* is the dominant sFlt-1 variant secreted by the endothelium [24]. We investigated whether the different generations of statins altered *sFlt-1 e15a or i13* mRNA expression in primary HUVECs. All statins significantly reduced *sFlt-1 e15a* expression (Fig. 1C). Simvastatin also significantly reduced *sFlt-1 i13* mRNA expression whilst pravastatin and rosuvastatin did not show any effect (Fig. 1D).

### Simvastatin is the most potent inhibitor of sFlt-1 secretion from primary trophoblasts and preeclamptic explants

**Fig. 2:**
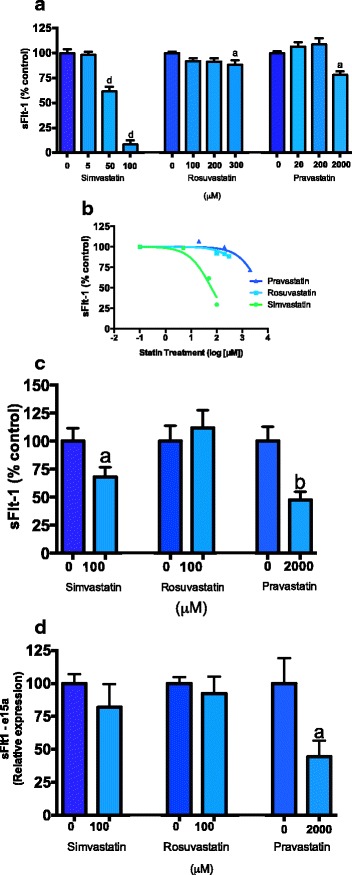
Effect of statins on sFlt-1 secretion and *sFlt-1 e15a* and *i13 mRNA* expression from placental tissues. **a** Simvastatin (0, 5, 50, 100 μM), rosuvastatin (0, 50, 100, 200, 300 μM) and pravastatin (0, 20, 200, 2000 μM) reduce sFlt-1 secretion from primary trophoblasts. **b** Inhibitory concentration of simvastatin, rosuvastatin and pravastatin was determined. **c** Simvastatin (100 μM) and pravastatin (2000 μM) significantly reduced sFlt-1 secretion from placental explants obtained from patients with preterm preeclampsia however rosuvastatin had no effect. **d** Pravastatin reduced *sFlt-1 e15a mRNA* expression from placental explants whilst simvastatin and rosuvastatin had no effect. Data represents *n* = 3-4 separate experiments and is expressed as mean ± SEM. *Dark blue bars* = control, *light blue bars* = statin treatments. a = *p* < 0.05, b = *p* < 0.01, d = *p* < 0.0001

We next compared the effect of the different statins on sFlt-1 secretion from primary trophoblasts. All statins significantly reduced sFlt-1 secretion from primary trophoblasts at top doses (Fig. 2A). Simvastatin was the most potent inhibitor of sFlt-1 secretion with an IC50 of 61.4 μM. It was 33 times more potent than rosuvastatin (IC50 2029 μM) and 85 times more potent than pravastatin (IC50 5224 μM) (Fig. 2B).

We also investigated the effects of the different statins on sFlt-1 secretion from placental explants obtained from patients with preterm preeclampsia (Fig. 2C). Simvastatin was a more potent inhibitor of sFlt-1 secretion whilst at the same dose rosuvastatin did not show any effect and pravastatin only showed an effect at 20 times this dose (Fig. 2C).

In the same preterm preeclamptic explants, simvastatin and rosuvastatin did not change *sFlt-1 e15a* expression, however pravastatin significantly reduced its expression at a dose of 2000 μM (Fig. 2D).

### All statins increase soluble endoglin (sENG) secretion from endothelial cells and do not affect secretion from placental explants

**Fig. 3:**
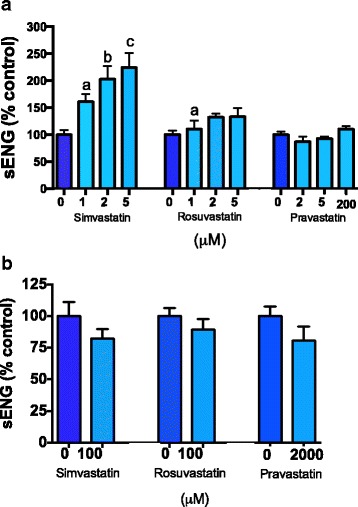
Effect of statins on soluble endoglin secretion from primary human tissues. **a** Simvastatin (0, 1, 2, 5 μM), rosuvastatin (0, 1, 2, 5, μM) and pravastatin (0, 2, 5, 200 μM) all caused an increase in soluble endoglin secretion from primary HUVECs. **b** Statin treatment did not alter sEng secretion from preterm preeclamptic explants. Data represents *n* = 3–4 separate experiments and is expressed as mean ± SEM. *Dark blue bars* = control, *light blue bars* = statin treatments. a = *p* < 0.05, b = *p* < 0.01, d = *p* < 0.0001

We next compared the effect of the statins on secretion of sENG from primary endothelial cells, and placental explants obtained from women with preterm preeclampsia. Previously we have shown that pravastatin significantly increases sENG secretion from endothelial cells [10], and here we show that simvastatin and rosuvastatin also increased its secretion. Simvastatin was the most potent inducer of sENG secretion followed by rosuvastatin and then pravastatin (Fig. 3A). In contrast, as previously demonstrated with pravastatin [10], there was no change in sENG secretion from placental explants from patients with preterm preeclampsia treated with the simvastatin or rosuvastatin (Fig. 3B).

### All statins upregulate heme-oxygenase 1 in primary endothelial cells

**Fig. 4:**
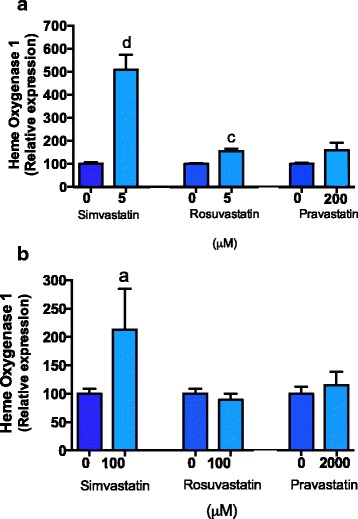
Effect of statins on heme-oxygenase 1 mRNA expression. **a** Simvastatin (0, 5 μM), rosuvastatin (0, 5 μM) and Pravastatin (0, 200 μM) up-regulated *heme-oxygenase 1* expression by HUVECs. **b** Simvastatin 100 μM up-regulated *heme-oxygenase 1* in preeclamptic placental explants, whist pravastatin 2000 μM and rosuvastatin 100 μM had no significant effect. Data represents *n* = 3–4 separate experiments and is expressed as mean ± SEM. *Dark blue bars* = control, *light blue bars* = statin treatments. a = *p* < 0.05, c = *p* < 0.001, d = *p* < 0.0001

Preeclampsia is thought to be associated with increased oxidative stress [3]. Therefore it would be beneficial if statins could up-regulate endogenous antioxidant defenses. We have previously shown pravastatin up-regulates heme-oxygenase 1 in endothelial cells however has no effect on its expression in placental explants [10]. Here we assessed whether simvastatin and rosuvastatin regulate *heme-oxygenase 1* expression in either endothelial cells or placental explants from women with preterm preeclampsia. Indeed, we found all statins increased *heme-oxygenase 1* mRNA expression in endothelial cells (Fig. 4A). Simvastatin significantly increased *heme-oxygenase 1* mRNA expression in placental explants taken from women with preterm preeclampsia (Fig. 4B) however rosuvastatin and pravastatin had no effect on its expression.

## Discussion

Statins have been proposed as possible therapeutics for preeclampsia. Our group [10] and Cudmore et al. [9], have shown pravastatin and simvastatin reduced sFlt-1 secretion from placental explants and endothelial cells respectively. The decrease in sFlt-1 caused by these statins was directly mediated through HMG CoA reductase [9, 10]. Pravastatin is the weakest inhibitor of HMG CoA reductase and it is conceivable that the more potent inhibitors of this enzyme (simvastatin or rosuvastatin) [17] may have a more pronounced effect on sFlt-1 secretion. Hence in this study, we examined the relative potency of three generations of statins on sFlt-1 secretion.

We found simvastatin was the most potent inhibitor of sFlt-1 secretion from all primary tissues examined. It was 28–85 times more potent than pravastatin and 3–33 times more potent than rosuvastatin at reducing sFlt-1 secretion from placental and endothelial cells respectively. Whilst simvastatin is known to be a more potent inhibitor of HMG CoA reductase than pravastatin, it is not as potent as rosuvastatin [17]. Perhaps the more pronounced effect of simvastatin on sFlt-1 secretion is a result of the hydrophobicity of the molecule. In contrast to pravastatin and rosuvastatin, simvastatin is hydrophobic and is readily able to cross the cellular lipid bilayer membrane [25, 26]. Therefore it is possible that this characteristic enhanced its capacity to enter the cells and reduce sFlt-1, relative to hydrophilic compounds, pravastatin and rosuvastatin. This effect was particularly apparent in endothelial cells, where simvastatin-induced a significant decrease in both *sFlt-1 e15a* and *sFlt-1 i13* isoforms, culminating in reduced sFlt-1 secretion. This is in contrast to rosuvastatin, which only reduced *sFlt-1 e15a*, and pravastatin that was only effective at reducing these variants at much higher doses [9, 10].

The effect of statins on the secretion of soluble endoglin is concerning. We previously demonstrated pravastatin up-regulates soluble endoglin secretion from endothelial cells [10]. Here we demonstrate that both simvastatin and rosuvastatin also upregulate soluble endoglin secretion from endothelial cells. Intriguingly, the effect on soluble endoglin appears also to relate to statin potency, with simvastatin inducing the most potent increase. Atorvastatin has previously been shown to upregulate membrane bound endoglin in endothelial cells, thus it is possible the increased soluble endoglin secretion we observed is a result of the same mechanism [27]. Reassuringly, we did not observe an increase in soluble endoglin secretion from preeclamptic explants treated with statins. Therefore, we conclude that the effect statins have on soluble endoglin secretion in vivo warrants further exploration and should be measured in future studies.

Of interest, the three statins studied herein had tissue specific effects on heme-oxygenase 1. Whilst robust up-regulation was observed in primary endothelial cells treated with all three classes of statins, only simvastatin increased heme-oxygenase 1 expression in placental explants obtained from women with preterm preeclampsia. This supports the premise that heme-oxygenase 1 may not regulate sFlt-1 secretion [28]; as pravastatin reduced sFlt-1 secretion with no change to heme-oxygeanse 1 expression in preterm preeclamptic placental explants [28]. Up-regulating an antioxidant molecule may be beneficial however and this further supports the beneficial effects of simvastatin as a possible preeclampsia therapeutic.

Although our data suggest simvastatin is the most potent statin at reducing sFlt-1 secretion, it is important to note that there have been concerns regarding its safety profile as a therapeutic for preeclampsia. This stems from the retrospective observational study performed by Edison [29] reporting an increase in limb defects in fetuses exposed to simvastatin in the first trimester. This finding has been challenged by other large retrospective observational studies [30, 31] showing no increased risk of fetal malformation in simvastatin exposed pregnancies compared to baseline. Furthermore, Gibb [32] questioned the validity of observations by Edison et al. [29] as there was no distinctive pattern to the limb defects described and the population baseline risk was not taken into account. As clinical onset of preeclampsia occurs in the second and third trimester after the majority of organogenesis has occurred, and the current treatment of preeclampsia consists solely of delivery, inflicting high rates of death and disability due to prematurity, we propose that the possible benefits of simvastatin administration may outweigh the theoretical risk.

## Conclusion

In conclusion, we have performed a direct comparison of three different generations of statins on key biochemical features of preeclampsia using primary human cells and tissues. Simvastatin was the most potent inhibitor of sFlt-1 secretion and the most potent agent in up-regulating heme-oxygenase 1 expression. Therefore, it is possible that it may be more efficacious as a treatment for preeclampsia compared to other statins.

## Abbreviations

IC50: inhibitory concentration 50
sENG: soluble endoglin
sFlt-1: soluble fms-like tyrosine kinase 1
